# Less is more: Enterobactin concentration dependency in copper tolerance and toxicity

**DOI:** 10.3389/fmolb.2022.961917

**Published:** 2022-08-16

**Authors:** Daiana Romina Peralta, Juan Vicente Farizano, Natalia Bulacio Gil, Natalia Soledad Corbalán, María Fernanda Pomares, Paula Andrea Vincent, Conrado Adler

**Affiliations:** Instituto Superior de Investigaciones Biológicas (INSIBIO), CONICET-UNT and Instituto de Química Biológica “Dr. Bernabé Bloj”, Facultad de Bioquímica, Química y Farmacia, UNT, Tucumán, Argentina

**Keywords:** copper, tolerance, toxicity, enterobactin, ROS-reactive oxygen species

## Abstract

The ability of siderophores to play roles beyond iron acquisition has been recently proven for many of them and evidence continues to grow. An earlier work showed that the siderophore enterobactin is able to increase copper toxicity by reducing Cu^2+^ to Cu^+^, a form of copper that is more toxic to cells. Copper toxicity is multifaceted. It involves the formation of reactive oxygen species (ROS), mismetallation of enzymes and possibly other mechanisms. Given that we previously reported on the capacity of enterobactin to alleviate oxidative stress caused by various stressors other than copper, we considered the possibility that the siderophore could play a dual role regarding copper toxicity. In this work, we show a bimodal effect of enterobactin on copper toxicity (protective and harmful) which depends on the siderophore concentration. We found that the absence of enterobactin rendered *Escherichia coli* cells more sensitive to copper, due to the reduced ability of those cells to cope with the metal-generated ROS. Consistently, addition of low concentrations of the siderophore had a protective effect by reducing ROS levels. We observed that in order to achieve this protection, enterobactin had to enter cells and be hydrolyzed in the cytoplasm. Further supporting the role of enterobactin in oxidative stress protection, we found that both oxygen and copper, induced the expression of the siderophore and also found that copper strongly counteracted the well-known downregulation effect of iron on enterobactin synthesis. Interestingly, when enterobactin was present in high concentrations, cells became particularly sensitive to copper most likely due to the Cu^2+^ to Cu^+^ reduction, which increased the metal toxicity leading to cell death.

## Introduction

Metals such as iron, manganese, magnesium, copper, and others are co-factors for several enzymes involved in a wide range of biochemical processes. Therefore, they constitute essential micronutrients required by microbes (Arguello et al., 2013). However, when intracellular concentrations surpass the physiological needs, they become highly toxic (Chandrangsu et al., 2017). Consequently, microorganisms have evolved several homeostatic mechanisms to tightly regulate intracellular metal levels. These mechanisms involve a balanced control of uptake, efflux and metal sequestration (Arguello et al., 2013; Ladomersky and Petris, 2015; Chandrangsu et al., 2017). In *E. coli,* copper requirements are in the nanomolar range (Nies and Herzberg, 2013) and only few enzymes use copper [i.e., copper SOD, SodC; NADH dehydrogenase-2, NDH-2; cytochrome oxidase, CytBO(3); aromatic amine oxidase, MaoA; and 3-deoxy-D-arabino-heptulosonate-7-phosphate synthase, AroF] (Rensing and Grass, 2003). Protein chaperones are supposed to transport copper to the recipient enzymes thus preventing the adventitious mismetallation and its associated toxicity (Rensing and McDevitt, 2013). Excess of copper disturbs the cellular redox potential and destabilize iron-sulfur clusters of dehydratases and other vital enzymes. It also leads to the formation of incorrect disulfide bonds and catalyzes the generation of highly reactive hydroxyl free radicals that readily damage biomolecules, such as DNA, lipids and proteins (Dupont et al., 2011). *E. coli*, encounters copper mainly in the gut of warm-blooded animals where concentrations can be as high as 10 µM (Rensing and Grass, 2003). Pathogenic bacteria may also face high Cu^+^ in the phagolysosome in macrophages (Stafford et al., 2013). Copper homeostasis in *E. coli* involves the P-type ATPase CopA that carries out excess of Cu^+^ from the cytoplasm to the periplasm (Rensing et al., 2000). Next, the multi-component CusCFBA system transports the metal out of the cell (Franke et al., 2003). Since copper reducing conditions increase the metal toxicity, (Macomber et al., 2007; Porcheron et al., 2013; Tan et al., 2017), another mechanism for copper detoxification consists in the oxidation of Cu^+^ to Cu^2+^ by means of the multicopper oxidase CueO (Grass and Rensing, 2001). Unlike copper efflux, which has been thoroughly studied and shown to require energy input to overturn the chemical gradient, copper uptake is poorly understood and would occur by a so far unknown copper importer (Giachino and Waldron, 2020). Interestingly, it has been demonstrated that certain siderophores can influence copper uptake (Johnstone and Nolan, 2015). For example, yersiniabactin by complexing copper may favor a controlled entry of this metal into the cell when it is required. However, when copper is in excess, the siderophore sequesters the metal outside bacterial cells protecting them (Koh et al., 2017; Robinson et al., 2018). This strategy of minimizing metal toxicity while retaining the nutritional access has been termed “nutritional passivation” (Koh et al., 2017). Another siderophore with an apparent dual behavior regarding copper toxicity is enterobactin. Enterobactin is a tri-catecholate siderophore, naturally produced by all *E. coli* strains as well as many other enterobacteria. It is the molecule with the highest affinity for iron reported so far (Khan et al., 2018). Under certain conditions, enterobactin takes part in different processes such as Cu^2+^ reduction to Cu^+^, with the concomitant increase in copper toxicity (Li et al., 1994; Schweigert et al., 2001; Chaturvedi et al., 2012) and also in oxidative stress reduction caused by various stressors (Adler et al., 2012; Adler et al., 2014; Peralta et al., 2016). As copper toxicity is mediated in part by oxidative stress, we considered of value to reexamine the role of enterobactin regarding copper homeostasis. In this work, we show that *E. coli* cells lacking enterobactin had increased copper sensitivity as a consequence of the higher reactive oxygen species (ROS) levels. Supporting the role of enterobactin as a ROS scavenger, we observed higher transcriptional expression and production of the siderophore in aerobic conditions compared with anaerobic ones. We also found that copper promoted enterobactin transcriptional expression, even in the presence of an excess of iron. In addition, we provide evidence indicating that in order to reduce copper-induced oxidative stress, enterobactin had to be internalized into the cell cytoplasm and then hydrolyzed by the esterase Fes. As expected, we observed that addition of enterobactin in relatively low concentrations lowered ROS levels along with cells sensitivity. Interestingly, media supplementation with higher concentrations of enterobactin led to cell death, most likely because of copper reduction. Together, our observations indicate that enterobactin would have a protective effect against copper toxicity, however when the siderophore concentrations rise to certain levels it has the opposite effect.

## Material and methods

### Bacterial strains and growth conditions

The *E. coli* K-12 strains and plasmids used in this study are described in Table 1. Bacterial cultures were grown in aerobic conditions at 37°C in M9 minimal salt medium (Sigma-Aldrich) supplemented with 0.2% casamino acids, 0.2% glucose, 1 mM MgSO_4_, and 1 mg/ml vitamin B1. Solid media contained 1.5% agar. For growth curves, culture aliquots were taken at different times and OD at 600 nm was measured. Anaerobic environments were generated in a Gaspak jar system using AnaeroGen sachets (OXOID, Sigma-Aldrich). When required, antibiotics (Kanamycin 50 μg/ml, Ampicilin 50 μg/ml) were added to media.

**TABLE 1:** List of strains and plasmids used in this work.

Strains	Relevant genotype	Source
*E. coli* BW25113	Wild-type	CGSC^a^
*E. coli* JW0586-1	BW25113 Δ*entE*::*kan*	CGSC^a^
*E. coli* CA10	BW25113 Δ*entE*::*lacZY*	Peralta et al. (2016)
*E. coli* JW0588-1	BW25113 Δ*entA*::*kan*	CGSC^a^
*E. coli* JW0587-1	BW25113 Δ*entB*::*kan*	CGSC^a^
*E. coli* JW0581-3	BW25113 Δ*fepG*::*kan*	CGSC^a^
*E. coli* JW0576-2	BW25113 Δ*fes*::*kan*	CGSC^a^
*E. coli* JW0584-1	BW25113 Δ*fepB*::*kan*	CGSC^a^
Plasmids		
pNTR-*entE*	pNTR-SD carrying *entE*	Saka et al. (2005)

a CGSC, *E. coli* genetic stock left.

### General methods

Plasmid DNA was isolated using the Wizard Miniprep DNA purification system (Promega) according to the manufacturer’s instructions. Transformation of competent cells using the CaCl_2_ procedure was performed as described previously (Sambrook et al., 1989).

### β-Galactosidase assays

The β-Galactosidase activities were determined following the method described by Zhou and Gottesman (1998), with a few modifications. Strain carrying the *entE::lacZY* transcriptional fusion was grown for 6 h in M9 medium at 37°C with aeration in presence or not (control) of 25 and 50 μM of CuSO_4_, FeCl_3_ or a combination of both metals, and assayed for β-Galactosidase activities. For this, 600 μl of these cultures were permeabilized with 0.1% SDS (24 μl) and chloroform (48 μl) for 20 min. Then, 100 μl of permeabilized cells were placed on 96-well microtiter plate and 100 μl of a 1.32 mg/ml solution of 2-nitrophenyl-β-D-galactopyranoside in buffer Z was added. Finally, the absorbance at 420 nm was measured for 20 min in a SpectraMax 250 spectrophotometer. Specific activity was calculated by dividing the slope of the line over time by the corresponding OD_600nm_ and expressed as arbitrary units (AU).

### Copper reducing power

*In vitro* copper reducing power of spent media obtained from different *E. coli* strains was measured using bathocuproine disulfonate (BCDS) (Sigma–Aldrich), following the method described by Volentini et al. (2011). This chelator forms a colored complex with cuprous ions, having a λmax at 480 nm (Poillon and Dawson, 1963). Cells were grown for 18 h at 37°C in M9 medium. Then, supernatants were filter sterilized and incubated with 100 μM CuSO_4_ and 200 μM BCDS. The absorbance at 480 nm was determined and Cu^+^ concentrations were calculated by comparison to a standard curve obtained in M9 medium supplemented with different Cu^2+^ concentrations and 25 mM ascorbic acid as a reducing agent.

### Copper sensitivity assays

Minimal inhibitory concentrations (MIC) were determined in M9 medium supplemented with 0.2% casamino acids, 0.2% glucose, 1 mM MgSO4 and 1 μg/ml vitamin B1. 5 μl of double dilutions from a 30 mM CuSO_4_ solution were spotted on M9 agar plates, dried and a lawn of the corresponding strain was overlaid. The plates were incubated at 37°C for 24 h under aerobic conditions or for 48 h under anaerobic conditions. MIC values were determined as the maximum dilution of CuSO_4_ that showed a zone of clearing. Enterobactin (ENT), catalase (CAT) and FeCl_3_ were used at concentrations of 1 μM, 1 mg/ml and 100 μM, respectively.

Cu-inhibition assays were performed using overnight cultures of the wild-type or the indicated mutant strains diluted 1:200 into fresh M9 medium supplemented with 0.2% casamino acids, 0.2% glucose, 1 mM MgSO_4_, 1 mg/ml vitamin B1 and containing either 0, 25, 50, or 100 μM of CuSO_4_. Experiments were done in a final volume of 10 ml. OD _600nm_ was followed over a 24 h-period of time for growth curves. For the evaluation of the impact of incremental concentrations of enterobactin on copper toxicity, overnight cultures of the *entE* strain were diluted 1:200 into fresh M9 medium supplemented with 0.2% casamino acids, 0.2% glucose, 1 mM MgSO4 and 1 mg/ml vitamin B1, containing 100 μM of CuSO_4_ and 0, 10, 20, or 50 μM of pure enterobactin. Experiments were done in a final volume of 200 μl. Cultures were incubated at 37°C with shaking, and bacterial viability as CFU/ml in LBA was determined at different times (0, 3, 18, and 24 h). At 24 h, Cu^+^ concentration was determined in the supernatants using BCDS (Sigma–Aldrich), following the method described above.

### Determination of catechol siderophore production

Catechol siderophore production was estimated according to the colorimetric method described by Arnow (1937). Briefly, cells were grown for 20 h at 37°C in M9 medium and supernatants filter sterilized. Then, 1 ml 0.5 N HCl, 1 ml nitrite-molybdate (1 g sodium nitrite and 1 g sodium molybdate dissolved in 10 ml water), 1 ml 1 N NaOH and 1 ml H_2_O were added to 1 ml of conditioned medium. Finally, the absorbance at 510 nm was determined and values were expressed as μM equivalents by comparison to a standard curve prepared using 2,3-dihydroxybenzoic acid (DHBA) (Sigma).

### Measurement of reactive oxygen species levels

ROS levels were measured using the oxidation-sensitive fluorescent dye 2,7-dichlorodihydrofluorescein diacetate (DCFH-DA). DCFH-DA is a cell-permeable dye widely used for the detection of ROS. This probe is enzymatically hydrolyzed by the action of cellular esterases to DCFH and then oxidized by the action of intracellular ROS (species such as hydrogen peroxide, hydroxyl radical, peroxyl radicals and nitrogen radicals) into 2′,7′-dichlorofluorescein (DCF), a molecule highly fluorescent that can be easily detected at 530 nm when excited at 485 nm (Gomes et al., 2005). Cells grown in M9 medium with or without 25 μM CuSO_4_ and 1 μM of pure enterobactin, were washed twice and resuspended in 50 mM sodium phosphate buffer, pH 7 at a final OD_600nm_ = 0.5. Then DCFH-DA was added at a final concentration of 10 μM and incubated for 30 min in darkness. After incubation, cells were washed, resuspended and sonicated in the same buffer. Fluorescence intensity was measured using a Perkin Elmer LS55 spectrofluorometer (excitation λ, 490 nm; emission λ, 519 nm) and results are expressed as relative fluorescence to that of the control.

### Statistical analysis

Results are expressed as the mean ± standard deviation (SD). The statistical analysis of the data was performed using the InfoStat statistical software (InfoStat versión 2020, http://www.infostat.com.ar). Analysis of variance (ANOVA) using a general linear model was applied to determine the effects of the factors evaluated in each analysis and significant differences (*p*-value < 0.05) between mean values were determined by Tukey’s test.

## Results

### Absence of enterobactin increases copper toxicity

**FIGURE 1:**
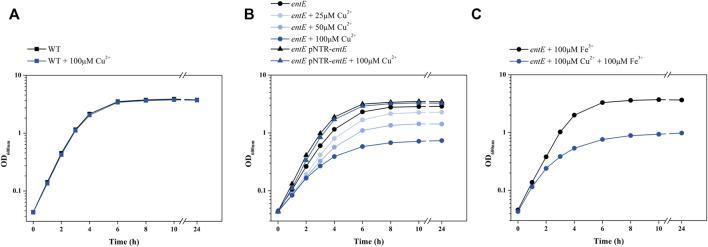
Effect of copper on *E. coli* growth. Growth curves obtained for *E. coli* wild-type (WT), *entE* mutant and *entE* mutant complemented with plasmid pNRT-*entE*. **(A)** Effect of copper addition on WT. **(B)** Effect of different copper concentrations on growth of *entE* and *entE* complemented with pNRT-*entE*. **(C)** Effect of simultaneous copper and iron supplementation on *entE* growth. Strains were cultured for 18 h in liquid aerated minimal M9 medium supplemented with 0, 25, 50, and 100 μM of CuSO_4_. Experiments were done at least in triplicates.

To test copper sensitivity in a condition with no enterobactin present, we made use of an *E. coli* strain harboring a mutation in the siderophore synthesis operon *entCEBA* (*E. coli entE*). Comparison of copper toxicity in *E. coli entE* and in its isogenic wild-type strain showed that wild-type growth in M9 medium was practically unaffected by supplementation with up to 100 μM CuSO_4_ (Figure 1A), while *entE* growth showed a dose-dependent inhibition response (Figure 1B). Interestingly, while iron addition restored *entE* growth to wild-type levels, it did not revert copper toxicity (Figure 1C). To rule out a pleiotropic effect caused by the mutation, we complemented *entE* strain with plasmid pNTR-*entE* and observed that the complemented strain showed an equivalent growth to that of the wild-type strain in all copper concentrations tested (25 and 50 µM is data not shown) (Figure 1A).

**FIGURE 2:**
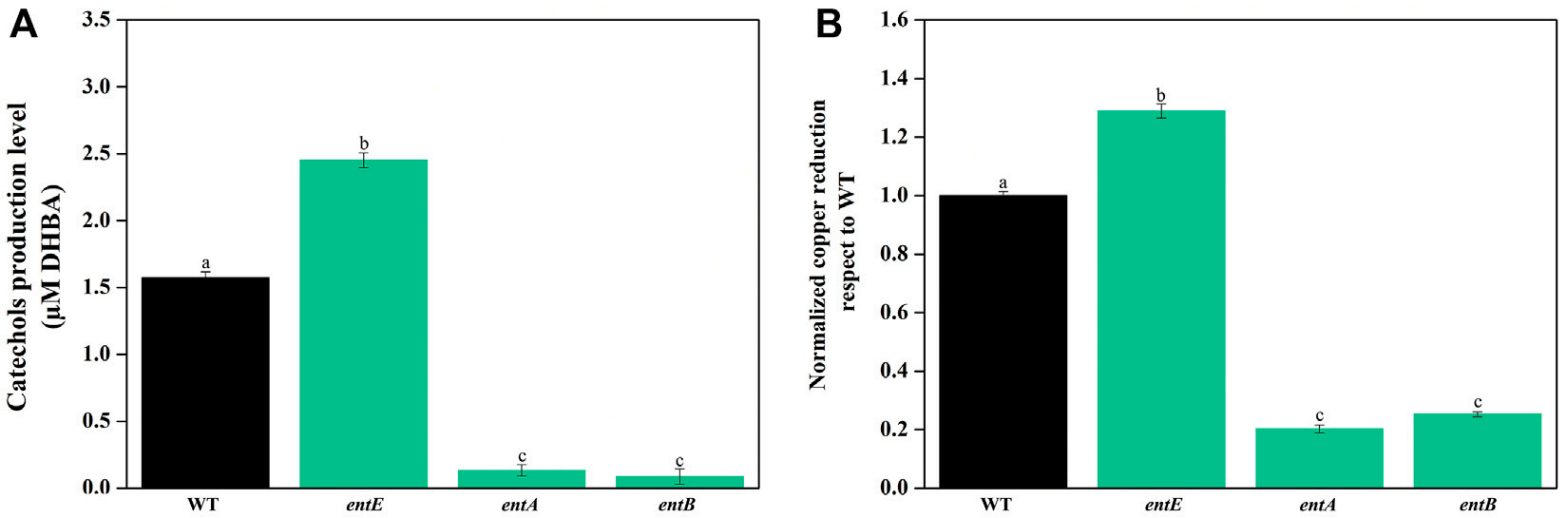
**(A)** Catechol content in supernatants of *E. coli* wild-type and mutants in the enterobactin system. Quantitation of catechol levels was performed using Arnow method with filter-sterilized supernatants obtained from wild-type (WT) and mutant strains impaired in enterobactin synthesis (*entE, entA*, and *entB*). Results are expressed as μM equivalents by comparison to a standard curve prepared using DHBA. Values are means ± SD for three independent experiments. **(B)** *In vitro* copper reducing power of supernatants of *E. coli* wild-type and mutants in the enterobactin system. Copper reducing activity was measured using bathocuproine disulfonate (BCDS). Values, normalized to Cu^+^ in WT supernatant, are the means ± SD of three independent experiments. Statistically significant differences between any two conditions compared are indicated with different letters (*p* < 0.05). Same letters indicate no statistically significant difference.

Next, we explored the possibility that the observed phenotype in the mutant strain was a consequence of an altered metabolic flux caused by the blockade in enterobactin biosynthesis pathway. Catechols found in wild-type strain supernatants are primarily enterobactin and biosynthetic intermediates in fewer amounts (Winkelmann et al., 1994). While disruption of enterobactin biosynthesis pathway in *entE* strain causes the absence of enterobactin and would favor the accumulation of the catechol precursor 2,3-dihydroxybenzoic acid (DHBA) (Cox et al., 1970; Pollack et al., 1970; Crosa and Walsh, 2002; Raymond et al., 2003; Ma and Payne, 2012), *entA* and *entB* mutants, by blocking enterobactin synthesis at an earlier biosynthesis step, produce no catecholate intermediates (Supplementary Figure S1). Thus, we analyzed whether there was a correlation between copper sensitivity and copper reduction capacity of supernatants having different catechol levels. Compared with wild-type, *entE* supernatant had higher catechol content and higher copper reducing ability (Figures 2A,B). As expected, *entA* and *entB* supernatants had negligible catechol content and consequently little copper reduction power which was about 5–7 fold lower compared to that of the *entE* strain (Figures 2A,B). Next, we evaluated copper sensitivity for the mutants (*entA*, *entB*, and *entE*) determining the copper minimal inhibitory concentration (MIC) for each strain. Table 2 shows that *entA* and *entB* strains had the same sensitivity to copper than *entE* strain which was significantly higher than that of the wild-type strain. Results thus far indicate that the increased sensitivity of mutants impaired in enterobactin synthesis stemmed from the lack of the siderophore and not from a pleiotropic effect or a metabolic shift due to the mutations.

**TABLE 2:** MIC of CuSO_4_ against selected bacterial strains with different additives and under anaerobic culture conditions.

	Culture conditions					
Strain	M9^a^	M9 + ENT^b^	M9 + FeCl_3_ ^c^	M9 + CAT^d^	M9-Anaerobic^e^	M9-Anaerobic + ENT^f^
Wild-type	30	30	30	N/D	7.5	3.75
*entE*	0.94	30	1.88	15	7.5	3.75
*entE* pNTR-*entE*	30	N/D	30	N/D	N/D	N/D
*entA*	0.94	30	1.88	N/D	N/D	N/D
*entB*	0.94	30	N/D	15	7.5	N/D
*fepB*	0.47	0.47	N/D	N/D	N/D	N/D
*fepG*	0.47	0.47	N/D	N/D	N/D	N/D
*fes*	0.94	0.94	N/D	N/D	N/D	N/D

MIC values are expressed in mM of CuSO_4_.

a M9 medium.

b M9 + ENT medium supplemented with 1 μM enterobactin.

c M9 + FeCl_3_ medium supplemented with 100 μM FeCl_3_.

d M9 + CAT medium supplemented with 100 μl of catalase 1 mg/ml.

e M9-Anaerobic incubation for 48 h.

f M9-Anaerobic incubation for 48 h with 1 μM enterobactin.

N/D-not determined.

### Enterobactin alleviates copper toxicity by reducing ROS

**FIGURE 3:**
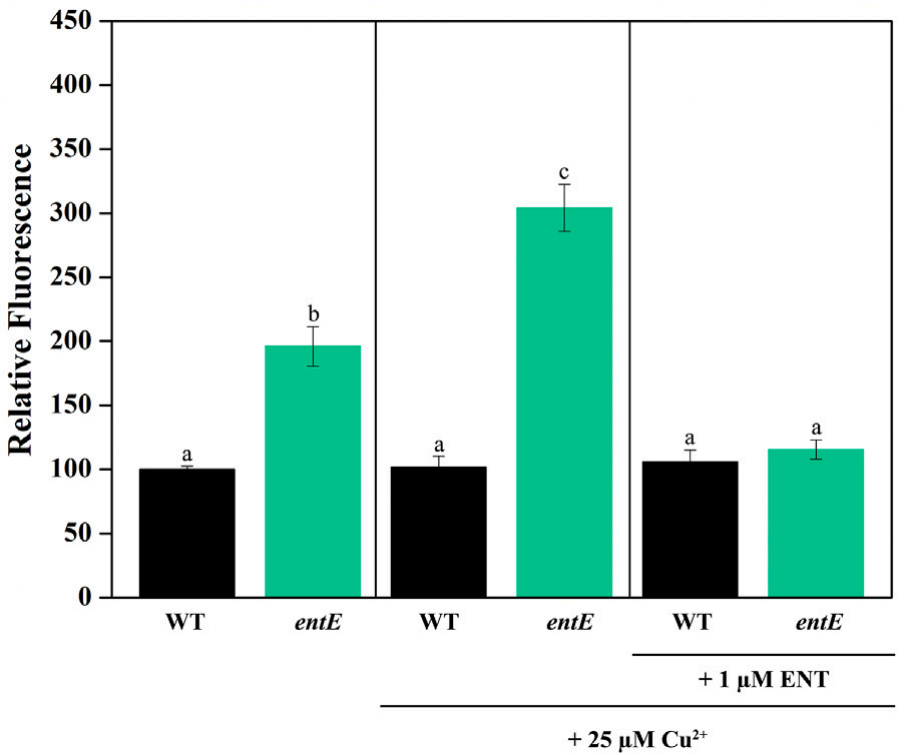
Measurement of ROS levels in *E. coli* wild-type and *entE* mutant. Impact of copper and enterobactin addition. Determination of ROS levels in wild-type (WT) and *entE* strains, grown in M9 with no copper added (left panel), with 25 µM of CuSO_4_ (central panel) and, with 25 µM CuSO_4_ plus 1 µM of enterobactin (right panel). Results are expressed as relative fluorescence to that of the control corresponding to WT strain grown in M9 medium. Values are means ± SD for three independent experiments. Statistically significant differences between any two conditions compared are indicated with different letters (*p* < 0.05). Same letters indicate no statistically significant difference.

To test the role of enterobactin in copper toxicity, we supplemented media with pure siderophore in a concentration of 1 μM which is in the range of the physiological concentration found in our experimental settings (Figure 2A). Table 2 shows that 1 μM of enterobactin added to *entE*, *entA*, and *entB* cultures rendered these strains equally sensitive to copper as the wild-type strain. Based on previous findings associating enterobactin with oxidative stress protection (Peralta et al., 2016) and the observation that iron supplementation did not reduce copper toxicity considerably (Table 2; Figure 1C), we assumed that enterobactin would protect against copper toxicity by reducing oxidative stress rather than facilitating iron uptake. Hence, we measured ROS levels in wild-type and *entE* strains in presence of copper and enterobactin. Equally to what we previously reported (Adler et al., 2014), *entE* strain grown in M9 with no copper or other stressor added, shows increased ROS levels compared with the isogenic strain (Figure 3 left panel). While medium supplementation with sublethal copper concentrations (25 μM of CuSO_4_) further increased ROS levels in the mutant strain, ROS levels remained constant in the wild-type (Figure 3 central panel). Then, when medium was supplemented with 25 μM of CuSO_4_ and 1 μM enterobactin, ROS levels dropped only for the *entE* mutant, reaching levels equal to those of the wild-type (Figure 3 right panel). Next, we tested the impact of medium supplementation with catalase on copper toxicity and found that the enzyme evenly reduced the sensitivity of *entE* and *entB* to CuSO_4_ (Table 2). To further characterize the role of enterobactin, we studied the siderophore effect on copper toxicity under anaerobic conditions. We found that in these conditions the wild-type, *entE* and *entB* strains had the same sensitivity to copper and that the addition of 1 μM enterobactin to the medium did not protect *entE* mutant from copper toxicity (Table 2). Our observations imply enterobactin in reducing copper toxicity only under aerobic conditions, being this consistent with the proposed role as a ROS scavenger.

### Enterobactin reduce copper toxicity after its uptake and hydrolysis

Previously, we demonstrated that in order to reduce the oxidative stress caused by pyochelin, paraquat or H_2_O_2_, enterobactin has to reach the cell cytoplasm and be hydrolyzed by the esterase Fes. Then, the hydroxyl groups in the catechol moieties are freed from iron coordination and are able to directly stabilize radicals (Peralta et al., 2016). We hypothesized that enterobactin, once linearized in the cell cytoplasm would also protect *E. coli* against copper-generated ROS. To address this hypothesis, we determined copper MIC for *E. coli* mutant strains in enterobactin uptake (*fepG* and *fepB*) and enterobactin hydrolysis (*fes*) in media supplemented with pure enterobactin. Table 2 shows that mutant strains *fepG*, *fepB* and *fes* were also significantly more sensitive to copper damage when compared to the isogenic wild-type strain. Unlike with strains impaired in enterobactin synthesis (*entA*, *entB*, and *entE*), addition of 1 μM enterobactin to the medium did not revert copper sensitivity of *fepG*, *fepB*, and *fes* mutants (Table 2). Consistently, addition of copper to *fepG* and *fes* cultures increased ROS levels and supplementation with 1 μM enterobactin did not reduce this increment (Supplementary Figure S2). As the increased copper toxicity of *fepG*, *fepB*, and *fes* mutants was correlated with augmented ROS and since both variables were unresponsive to enterobactin addition, the hydrolyzed form of the siderophore results as the candidate molecule responsible for ROS reduction.

### Oxidative stress-generating conditions induce enterobactin transcriptional expression

**FIGURE 4:**
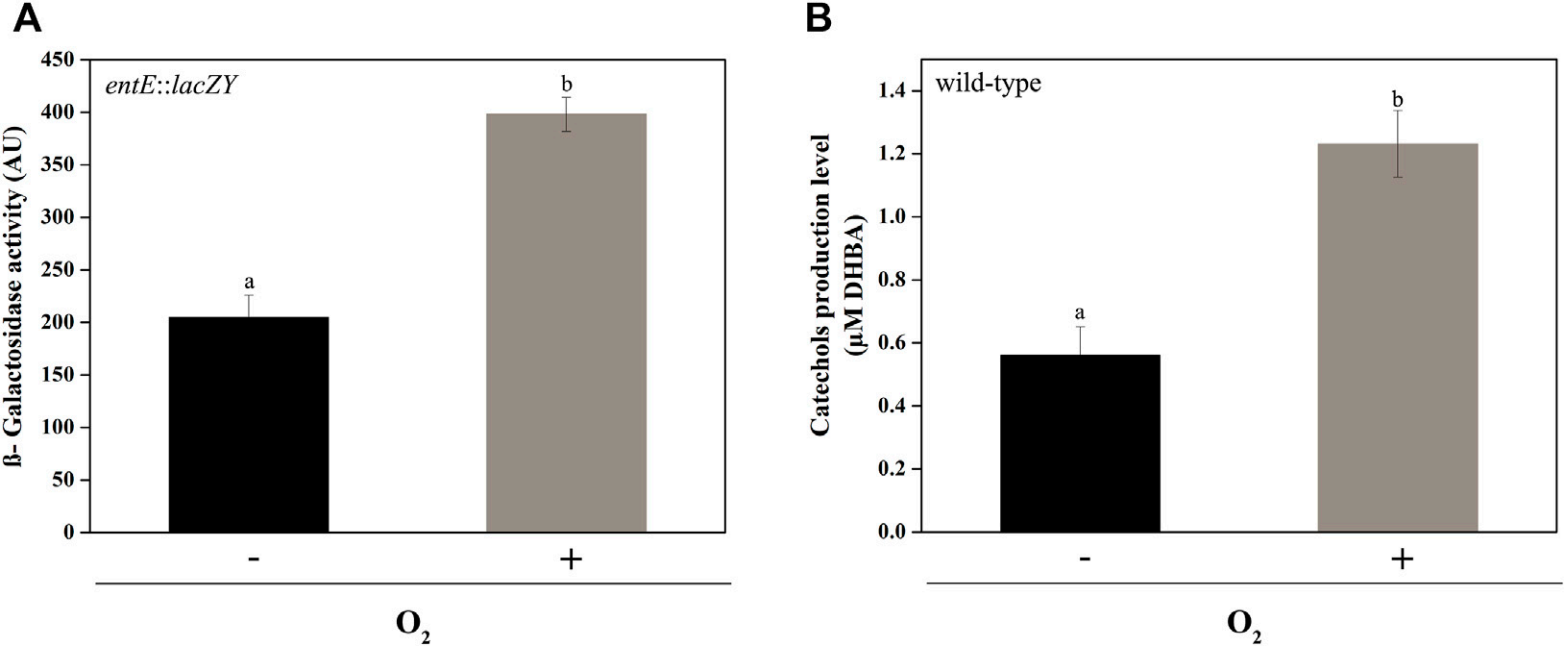
Comparison of *entE* expression and catechol production in aerobic and anaerobic culture conditions. **(A)** *entE* expression in aerobic and anaerobic culture conditions determined by means of β−Galactosidase assay using a strain with an *entE::lacZY* chromosomal fusion. **(B)** Determination of catechol production level in supernatants of *E. coli* wild-type in aerobic culture conditions compared with anaerobic ones. Quantitation was done using Arnow assay with filter-sterilized supernatants. Results are expressed as μM equivalents by comparison to a standard curve prepared using DHBA. Values are means ± SD for three independent experiments. Statistically significant differences between any two conditions compared are indicated with different letters (*p* < 0.05). Same letters indicate no statistically significant difference.

**FIGURE 5:**
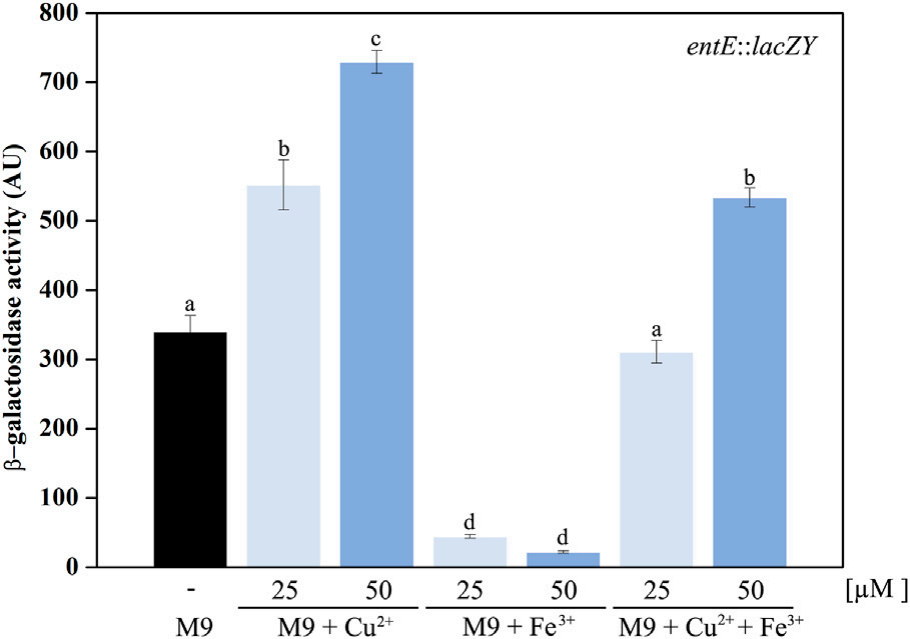
Induction of enterobactin transcriptional expression by copper. A strain with a chromosomal *entE::lacZY* transcriptional fusion was used as an indicator of enterobactin transcriptional expression. Data shows the control condition without CuSO_4_ or FeCl_3_ addition (black bar) and the effect of supplementing media with 25 and 50 µM CuSO_4_, 25 and 50 µM FeCl_3_ and simultaneous supplementation with both copper and iron. *entE* expression was determined by means of a β-Galactosidase assay described in the material and methods section. Values are means ± SD for three independent experiments. Statistically significant differences between any two conditions compared are indicated with different letters (*p* < 0.05). Same letters indicate no statistically significant difference.

Regulation of enterobactin synthesis by oxidative stress would be consistent with its proposed antioxidant role (Adler et al., 2014; Peralta et al., 2016). In fact, in this work we show that the *entE::lacZY* chromosomal fusion activity (Figure 4A) and the catechol content in supernatants of the wild-type strain (Figure 4B), were significantly higher in aerobic culture conditions compared with anaerobic ones. Next, we wondered whether copper would also be able to induce enterobactin expression. For this, we used the same strain with the chromosomal *entE*::*lacZY* transcriptional fusion and grew it in M9 medium with the addition of CuSO_4_ (see materials and methods). Compared with the control with no copper added, medium supplementation with 25 μM or 50 μM of CuSO_4_ significantly increased *entE* transcriptional expression in a dose-dependent manner (Figure 5). As expected, addition of 25 μM or 50 μM of FeCl_3_ with no copper added, highly repressed the expression of enterobactin (Figure 5). However, when medium was supplemented with both metals (copper and iron), *entE* transcription was only repressed partially (Figure 5). By measuring the activity of the Fur regulated promoter *ryhB* in the *entE* strain, we proved that iron availability was not affected by the addition of copper to the medium (data not shown). Evidence showed that aerobic culture conditions and copper, triggered enterobactin transcription thus further supporting the involvement of the siderophore in oxidative stress protection.

### Enterobactin concentration dependency in copper toxicity

After establishing that physiological concentrations of enterobactin, at least those found in our experimental conditions (Figure 2), protected against oxidative stress (Table 2; Figure 3), we decided to perform a dose-response curve for enterobactin concentrations in presence of copper. For that, we measured CFU/mL at different time points of *entE* cultures supplemented with 100 μM CuSO_4_ and with increasing enterobactin concentrations (1, 10, 20, and 50 μM enterobactin). As controls, we used *entE* cultures with no enterobactin added in the presence and absence of copper.

**FIGURE 6:**
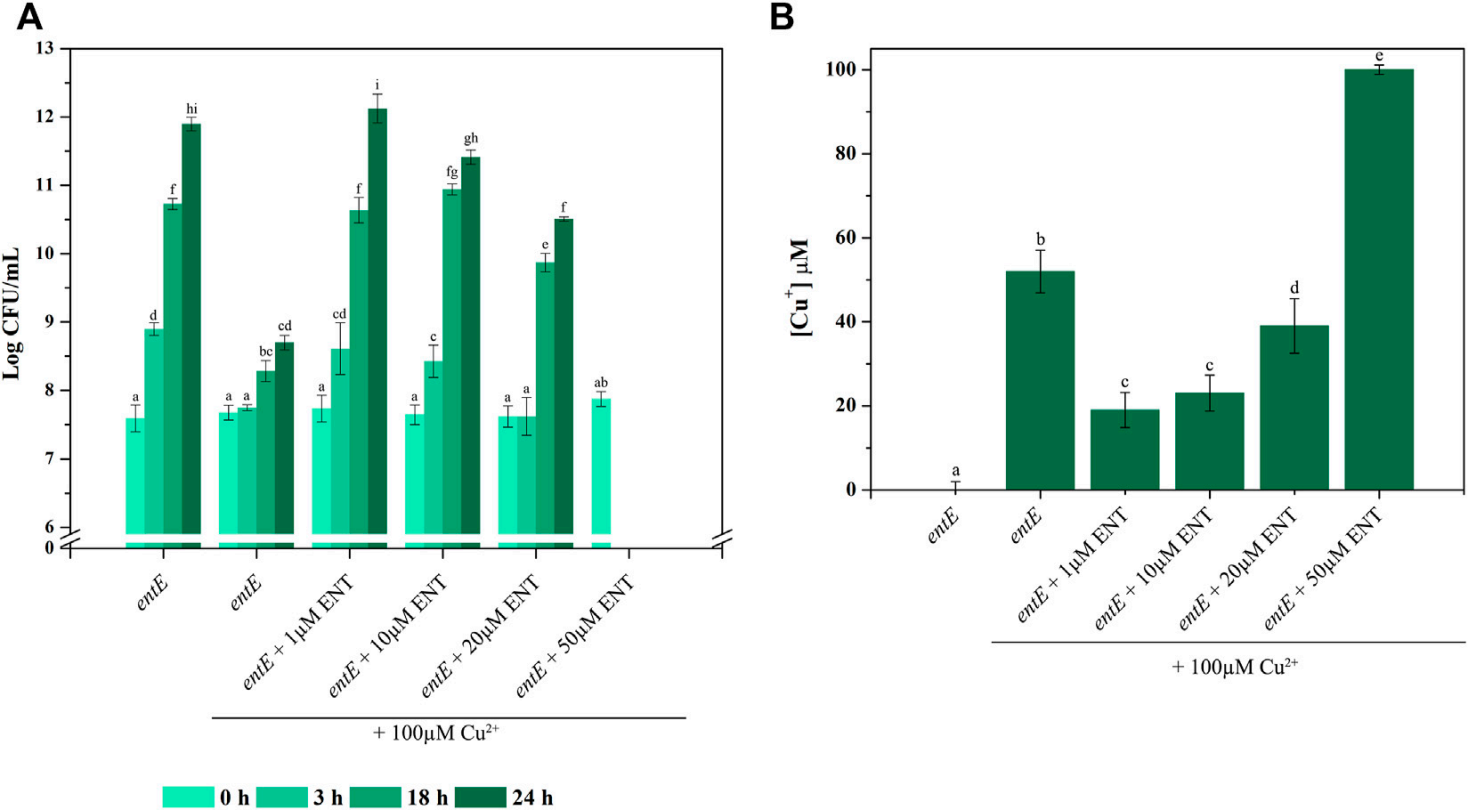
Concentration dependent effect of enterobactin on copper toxicity. **(A)** CFU/ml at different times (0, 3, 18, and 24 h) for *E. coli entE* strain grown in liquid aerated minimal M9 medium supplemented with 0, 1, 10, 20, and 50 µM of enterobactin (ENT) in presence of 100 µM of CuSO_4_. Data is plotted as the mean values ± SD of Log CFU/ml. Experiments were done in triplicates. **(B)** Cu^+^ concentration was determined by means of BCDS as described in material and methods. Data is plotted as the mean values ± SD of Cu^+^ concentrations determined at the end of the assay (24 h). Experiments were done in triplicates. Statistically significant differences between any two conditions compared are indicated with different letters (*p* < 0.05). Same letters indicate no statistically significant difference.

As observed before (Figure 1), *entE* mutant with no enterobactin added showed higher copper sensitivity (log CFU/mL at 24 h of 11.89 vs. 8.69 for *entE* and *entE* + 100 μM CuSO_4_, respectively) (Figure 6A). When enterobactin was added in relatively low concentrations (1–10 μM), copper toxicity was clearly reduced at all time points measured. Medium enterobactin concentration (20 μM) still protected from copper toxicity at 18 and 24 h but to a lesser extent compared with 1 and 10 μM enterobactin. Finally, a high enterobactin concentration (50 μM) resulted radically harmful for *E. coli* cells (Figure 6A). As a control for this condition, we supplemented media with 50 µM enterobactin with no addition of copper and found an *entE* strain growth of 11.75 log CFU/ml at 24 h. Analysis of Cu^+^ levels in supernatants of 24 h old cultures, revealed a substantial increment of Cu^+^ in the condition having 50 μM of enterobactin compared with no enterobactin or 1, 10, or 20 μM enterobactin (Figure 6B). It is likely that low enterobactin concentrations protected cells from copper toxicity by reducing ROS and that higher concentrations of the siderophore were detrimental to cells because of the Cu^2+^ to Cu^+^ reduction which increases copper toxicity.

## Discussion

We previously reported that the siderophore enterobactin protects *E. coli* against the oxidative stress generated by various stressors such as the Pseudomonad siderophore pyochelin, H_2_O_2_, and paraquat (Adler et al., 2012; Peralta et al., 2016). Given that copper involves the formation of ROS through Fenton-like reactions (Dupont et al., 2011), it is conceivable that enterobactin could play a role in copper tolerance. However, literature also implies enterobactin in reducing Cu^2+^ to Cu^+^ and therefore in increasing its toxicity (Chaturvedi et al., 2012). Thus, we considered necessary to evaluate *E. coli* copper sensitivity in conditions involving either no enterobactin, enterobactin in physiological levels and in increasing concentrations.

Our results clearly show that an *E. coli* strain unable to synthesize enterobactin (*entE* mutant) is substantially more susceptible to copper toxicity than the isogenic strain able to produce the siderophore (wild-type strain) (Figure 1). A pleiotropic effect in *entE* strain was ruled out by complementing the strain with plasmid pNTR-*entE* (Figure 1A; Table 2). The lack of correlation between the accumulation of catechols in mutants tested along with the copper reducing power and copper susceptibility, excluded the metabolic shift hypothesis (Figures 2A,B; Table 2). These observations further support that the deficiency of the siderophore in *entE* mutant is the underlying cause of increased copper toxicity. This susceptibility to copper was found to be dose dependent (Figure 1B) and not reverted by iron supplementation (Figure 1C; Table 2). Copper can be toxic to cells by generating oxidative stress and by displacing iron from Fe–S clusters. This causes and altered cellular redox potential and instability, inadequate conformation and malfunction of several vital iron-sulfur proteins (Macomber and Imlay, 2009). Hence, if increased copper toxicity in enterobactin deficient cells was due to iron displacement from Fe-S clusters, iron supplementation should have mitigated this effect. As addition of 100 µM FeCl_3_ to *entE* strain cultures did not revert the effect of 100 μM CuSO_4_ (Figure 1C; Table 2), we concluded that the lack of the siderophore did not lead to increased copper toxicity as a consequence of iron shortage.

Next, we evaluated the involvement of oxidative stress in the increased sensitivity to copper in cells lacking enterobactin. For that, we used the DCFH-DA probe which detects ROS, mainly hydrogen peroxide due to its higher stability (Kim and Xue, 2020). While the wild-type strain was able to maintain ROS levels constant upon copper supplementation, ROS levels increased in *entE* mutant*,* thus indicating that the strain was defective in dealing with the oxidative stress triggered by copper (Figure 3). Addition of 1 μM enterobactin decreased ROS in the *entE* strain to wild-type levels, corroborating the ability of the siderophore to reduce the oxidative stress (Figure 3). Moreover, addition of catalase, which alleviates oxidative stress derived from Fenton chemistry, significantly reduced the sensitivity of *entE* and *entB* strains (Table 2). Catalase decomposes hydrogen peroxide. Then, its ability to reduce cells sensitivity to copper indicates the relevance of this ROS species in the conditions tested. Furthermore, there was no difference in copper sensitivity for the wild-type and *entE* strains when they were cultured in anaerobic conditions either with no enterobactin added or with a 1 μM enterobactin supplementation (Table 2). These results further imply enterobactin in reducing oxidative stress caused by copper.

Previously, we reported on the requirement of enterobactin uptake and hydrolysis for the reduction of oxidative stress triggered by stressors other than copper (Peralta et al., 2016). In this work, we show that strains impaired in enterobactin uptake and hydrolysis (*fepG* and *fes*), despite being able to produce the siderophore, had also increased copper sensitivity (Table 2) and higher ROS levels (Supplementary Figure S2) compared with the wild-type. Given that these mutants were unresponsive to enterobactin addition, it can be inferred that the hydrolyzed form of the siderophore is the molecule that scavenges ROS. This is in concordance with our previous results and with a recent report showing that enterobactin protects *Salmonella* cells from hydrogen peroxide after being linearized and exported to the extracellular compartment (Bogomolnaya et al., 2020). Casanova-Hampton et al. (2021) also linked the enterobactin synthesis and uptake system with copper tolerance. In that work, authors speculated that the increased copper toxicity found in the enterobactin system mutants could result from an impairment in iron homeostasis. However, in this work we provide evidence that iron shortage would not be accountable for the increased sensitivity and that the inability to cope with oxidative stress would be the underlying cause. Our results indicate that enterobactin would play a significant role in copper toxicity protection only under aerobic conditions where oxygen is available to form ROS. Hence, upregulation of enterobactin production in such conditions would be expected.

In fact, the analysis of *entE::lacZY* expression and catechol content in supernatants showed that these two variables were higher under aerobic conditions compared with anaerobic ones (Figures 4A,B). In addition, copper promoted the *entE* transcriptional expression in a dose dependent manner and counteracted the classical repression mediated by iron (Figure 5) (Hunt et al., 1994; Christoffersen et al., 2001). These results clearly show the relevance of the siderophore in conditions implying oxidative stress. Stressors such as paraquat and hydrogen peroxide were previously shown to indirectly induce enterobactin expression through ROS and probably involving the regulon *soxRS* (Peralta et al., 2016). Then, it is possible to assume that copper would act in a similar fashion (Kimura and Nishioka, 1997; Kershaw et al., 2005; Yamamoto and Ishihama, 2005). In agreement with this, the gene expression profile of *E. coli* MG1655 analyzed by oligonucleotide microarray showed that genes taking part in the biosynthesis and uptake of enterobactin are increased in *E. coli* cells when these are exposed to copper (Kershaw et al., 2005). Furthermore, Casanova-Hampton et al. (2021) recently showed that transcription of genes involved in the biosynthesis and uptake of enterobactin were significantly upregulated in *E. coli* during copper stress. Similarly, a microarray-based comparative analysis of the transcriptional response of *Salmonella* Typhimurium to copper, showed the transcriptional induction of enterobactin synthesis operon (*entCEBA*) upon exposure to copper (Pontel et al., 2014).

**FIGURE 7:**
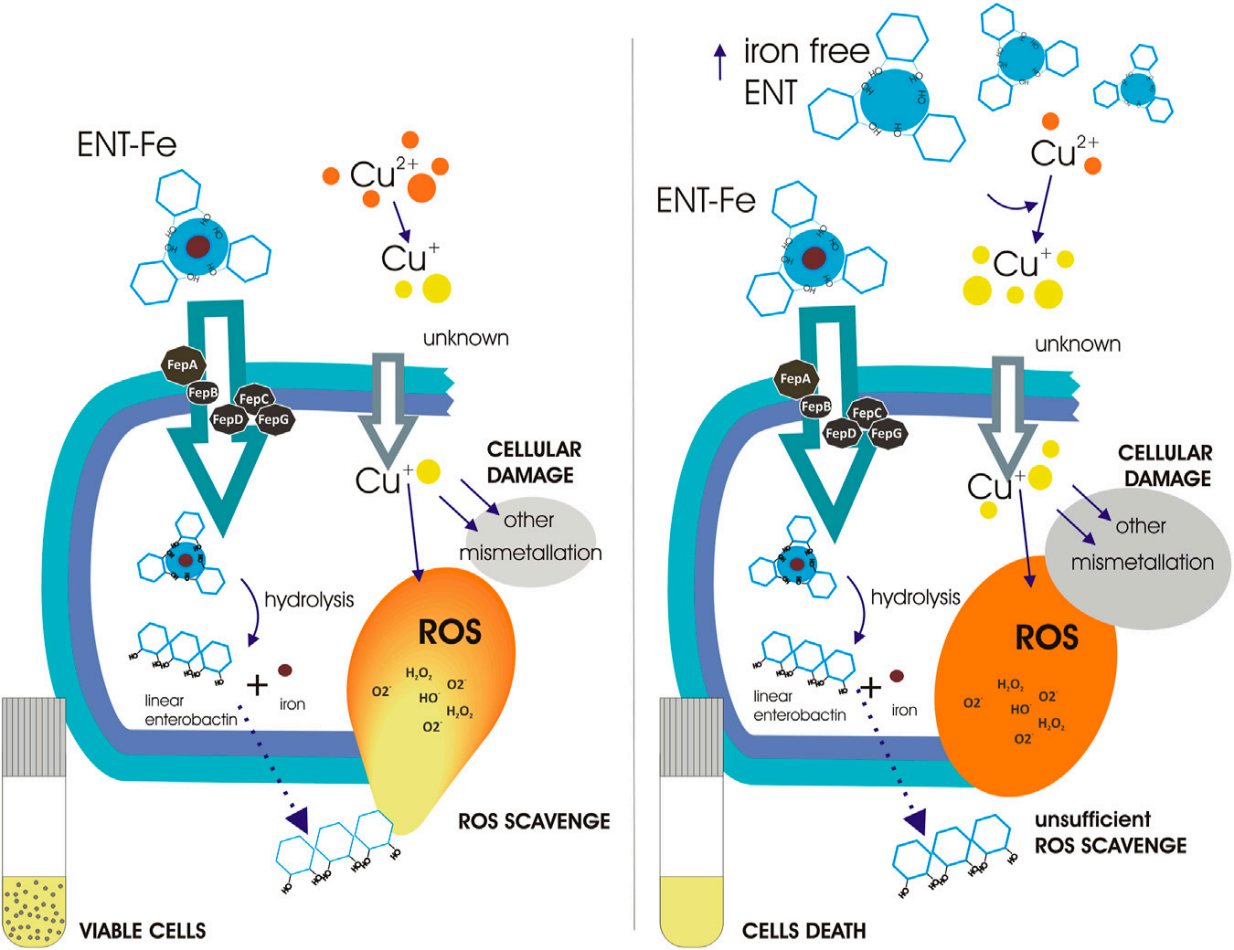
Schematic representation of the proposed role of enterobactin in copper tolerance and toxicity. Enterobactin enters cells as an iron-enterobactin complex. Uptake requires of the transport proteins FepA, FepB, FepC, FepD, and FepG. Once in the cytoplasm, the enterobactin-iron complex is hydrolyzed by Fes leaving enterobactin as a linear molecule, which is able to reduce ROS, and free iron for cellular metabolism. Linear enterobactin is possibly exported to the extracellular space of *E. coli*, where it would detoxify extracellular ROS, similarly as was recently reported for *Salmonella* (Bogomolnaya et al., 2020). Left panel shows that low enterobactin concentrations help cells to cope with copper-induced ROS. Even though, copper toxicity is multifaceted, enterobactin protection allows cells survival. Right panel when enterobactin is found in high concentrations, its ability to reduce Cu^2+^ to Cu^+^ enhances copper toxicity leading to cell death. The prevailing toxic mechanism of copper is unknown in this situation.

Finally, the impact of increasing enterobactin concentrations on copper toxicity was evaluated following CFU/ml over time. Figure 6A shows that medium supplementation with enterobactin in a range of 1–20 μM, protected cells against copper toxicity. This protection peaked at 1 μM and then slightly diminished as enterobactin concentration increased up to 20 μM. When 50 μM enterobactin was used, a dramatic change in CFU/ml was observed. The decrease in the protection against copper toxicity as enterobactin increased its concentration is inversely correlated with Cu^+^ found in supernatants (Figure 6B). Since medium supplementation with 50 μM enterobactin, with no copper added, did not negatively affect *entE* strain growth, we assume that the dramatic cell death observed at 50 μM enterobactin plus copper, resulted from the increased copper toxicity due to the higher presence of Cu^+^. Thus, it is likely that at low enterobactin concentrations, the ability of the siderophore to reduce the oxidative stress after its uptake and hydrolysis leads to less copper toxicity. However, as enterobactin concentration is augmented, the surplus of the siderophore is probably available for Cu^2+^ reduction to Cu^+^, hence favoring its toxicity. The balance between these two opposing effects would depend on the siderophore concentration, being the critical point between 20 and 50 μM. This level of enterobactin is substantially higher to that found in our experimental conditions and therefore it raises the question whether this situation would reflect an actual physiological condition. However, we cannot rule out a natural situation implying higher enterobactin levels leading to a detrimental effect if high copper concentrations are present. A simplified model of the proposed role of enterobactin in copper tolerance and toxicity in *E. coli*, is presented in Figure 7. To conclude, as enterobactin producing strains (intestinal commensals, intestinal pathogens, uropathogenic strains, free-living strains, etc.) have substantial genetic background differences, including the ability to produce other siderophores, and grow in dissimilar environments, we find the necessity of a broader study, covering such differences, in order to get a clearer picture of the biological relevance of enterobactin.

## Data Availability

The original contributions presented in the study are included in the article/Supplementary Material, further inquiries can be directed to the corresponding authors.
