# Supplementary material for: Less is more: Enterobactin concentration dependency in copper tolerance and toxicity
Source: Front Mol Biosci. 2022 Aug 16;9:961917. doi: 10.3389/fmolb.2022.961917 (PMC9426971; doi:10.3389/fmolb.2022.961917)
Supplement: Supplementary file 1 [file Image1.pdf]

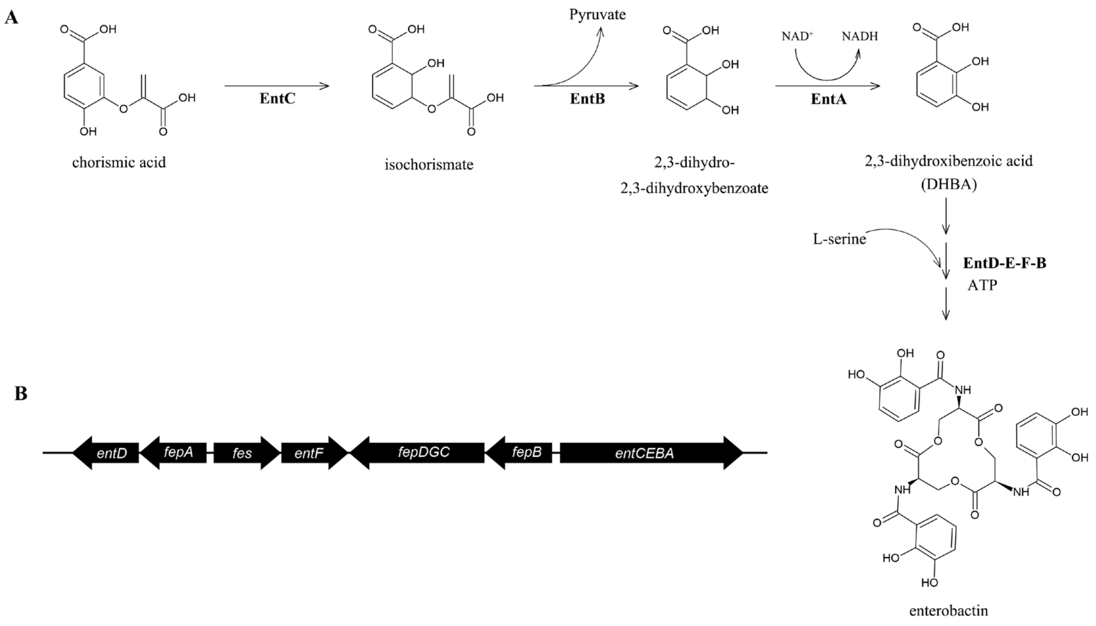

Figure S1. A) Enterobactin biosynthetic pathway in *Escherichia coli.* B) Enterobactin gene cluster including synthesis and uptake operons.

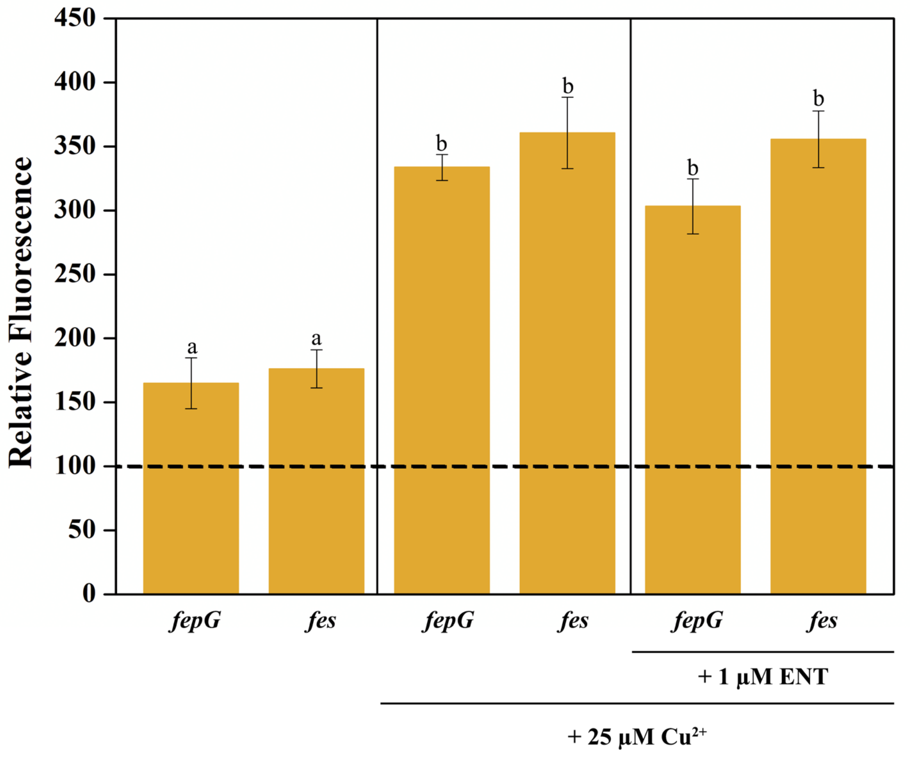

Figure S2. Measurement of reactive oxygen species levels in *E. coli fepG* and *fes* strains. Impact of copper and enterobactin addition. Determination of reactive oxygen

species (ROS) levels in *E. coli fepG* and *fes* strains, grown in M9 with no copper added (left panel), with 25 μM of $CuSO_4$ (central panel) and, with 25 μM $CuSO_4$ plus 1 μM of enterobactin (right panel). Results are expressed as relative fluorescence to that of the control corresponding to WT strain (dotted line) grown in M9 medium. Values are means ± SD for three independent experiments. Statistically significant differences between any two conditions compared are indicated with different letters ($P<0.05$). Same letters indicate no statistically significant difference.
